# Janus kinase inhibition suppresses PKC-induced cytokine release without affecting HIV-1 latency reversal ex vivo

**DOI:** 10.1186/s12977-016-0319-0

**Published:** 2016-12-20

**Authors:** Adam M. Spivak, Erin T. Larragoite, McKenna L. Coletti, Amanda B. Macedo, Laura J. Martins, Alberto Bosque, Vicente Planelles

**Affiliations:** 1 Department of Medicine, University of Utah School of Medicine, Salt Lake City, UT USA; 2 Department of Pathology, University of Utah School of Medicine, Emma Eccles Jones Medical Research Building Room 2520, 15 North Medical Drive East, Salt Lake City, UT 84112 USA

**Keywords:** HIV, Viral latency, Ingenol, Janus kinase inhibition, Ruxolitinib

## Abstract

**Background:**

Despite the durable viral suppression afforded by antiretroviral therapy, HIV-1 eradication will require strategies to target latently infected cells that persist in infected individuals. Protein kinase C (PKC) activation is a promising strategy to reactivate latent proviruses and allow for subsequent recognition and clearance of infected cells by the immune system. Ingenol derivatives are PKC agonists that induce latency reversal but also lead to T cell activation and the release of pro-inflammatory cytokines, which would be undesirable in vivo. In this work, we sought to identify compounds that would suppress pro-inflammatory cytokine production in the context of PKC activation.

**Design and methods:**

We performed an in vitro screen to identify compounds that could dampen pro-inflammatory cytokine release associated with T cell activation, using IL-6 as a model cytokine. We then tested the ability of the most promising screening hit, the FDA-approved Janus Kinase (JAK) inhibitor ruxolitinib, to diminish release of multiple cytokines and its effect on latency reversal using cells from HIV-1-positive, aviremic participants.

**Results:**

We demonstrate that co-administration of ruxolitinib with ingenol-3,20-dibenzoate significantly reduces pro-inflammatory cytokine release without impairing latency reversal ex vivo.

**Conclusion:**

The combination of ingenol compounds and JAK inhibition represents a novel strategy for HIV-1 eradication.

**Electronic supplementary material:**

The online version of this article (doi:10.1186/s12977-016-0319-0) contains supplementary material, which is available to authorized users.

## Background

Antiretroviral therapy (ART) blocks HIV-1 replication and allows for restoration of the circulating CD4^+^ T cell population in infected patients. However the virus persists in long-lived cellular reservoirs [1–3]. While ART can continuously suppress viral replication for years or even decades, patients who stop therapy soon develop viremia and progress to overt immunodeficiency if ART is not restarted [4]. Cells harboring this transcriptionally silent but inducible viral reservoir lack specific markers that would allow direct targeting in vivo. This has informed a pharmacologic eradication strategy making use of compounds to reverse the latent viral state and ‘unmask’ this reservoir [5]. HIV-1 infected cells could then be recognized and cleared via viral cytopathic effects or immune-mediated mechanisms, resulting in prolonged ART-free virologic remission. Several classes of latency-reversing agents (LRAs) have reached pilot clinical trials [6–11]. While LRAs have generally been well tolerated by study participants, to date no significant change in latent reservoir size has been observed in these trials.

Protein kinase C (PKC) agonists are a promising LRA class [12]. Ingenol derivatives appear more efficacious and less toxic in vitro than more widely studied PKC agonists such as prostratin or bryostatin-1 [13–15]. Ingenol mebutate is FDA approved as a topical therapy for actinic keratosis [16], and other ingenol compounds have had a wide variety of applications in traditional medicine [17]. We recently described the efficacy of ingenol-3,20-dibenzoate, a PKC agonist isolated from *Euphorbia* plant species, to induce viral transcription ex vivo in resting CD4^+^ T cells from HIV-1 infected patients [18]. Recent studies have identified the efficacy of PKC agonists including bryostatin-1 and ingenol derivatives in combination with LRAs from other mechanistic classes in vitro [12, 19–21] as well as in vivo in a non-human primate model [22]. Activation of NF-kB signaling is thought to be the mechanism by which PKC agonists reactivate latent HIV-1 provirus [23, 24]. Cellular PKC isoforms activate transcription factors including NF-kB, AP-1 and NF-AT leading to T cell activation [25–28]. Through these same pathways however, some PKC agonists can induce pro-inflammatory cytokine secretion [29, 30]. This could cause significant morbidity in vivo and has precluded PKC activation as a viable latency reversal strategy in clinical trials to date.

One strategy to address cytokine release associated with PKC activation would be the addition of a second pharmacologic agent to attenuate a pro-inflammatory response. In the present study we hypothesized that select kinase inhibitors could be identified which would dampen PKC-induced pro-inflammatory cytokine secretion. Our ultimate goal was to identify means of decreasing cytokine release while preserving the LRA properties of PKC agonists. Our unbiased in vitro screen identified ruxolitinib, an FDA-approved drug targeting the Janus kinase–signal transducer and activator of transcription (JAK–STAT) pathway. FDA-approved JAK inhibitors efficiently block pro-inflammatory cytokine release from T cells in vivo in the context of myelofibrosis [31] and rheumatoid arthritis [32].

This strategy has not been previously explored in the context of HIV eradication and represents a novel approach to access the potential of PKC activation in the clinic. Here we demonstrate that JAK inhibition using the FDA-approved drug ruxolitinib is capable of decreasing ingenol-induced pro-inflammatory cytokine release without significantly reducing latency reversal in resting CD4^+^ T cells from aviremic HIV-1 positive patients on ART.

## Methods

### Participants

Healthy donors and aviremic HIV-1 infected patients on ART were recruited for phlebotomy according to two approved Institutional Review Board (IRB) protocols at the University of Utah as described previously [18]. Inclusion criteria for HIV-1 infected participants required viral suppression (less than 50 HIV-1 RNA copies/mL) for a minimum of 6 months, ART initiation during chronic HIV-1 infection (>6 months since seroconversion), and compliance with a stable ART regimen for a minimum of 12 months per participant and provider report. Informed consent and phlebotomy were performed in the Center for Clinical and Translational Science Clinical Services Core at the University of Utah Medical Center.

### Reagents

Bryostatin-1, prostratin, ingenol-3,20-dibenzoate and ingenol-3-hexanoate, also known as ingenol B, were obtained from the Martin Delaney Collaboratory of AIDS Researchers for Eradication (CARE) Pharmacology Core, University of North Carolina, Chapel Hill, NC. The kinase inhibitor library was obtained from the University of Utah Drug Discovery Core Facility. CD3/CD28 antibody-coated magnetic beads (Dynabeads^®^ Human T-Activator CD3/CD28) were purchased from Life Technologies (ThermoFisher Scientific). Ruxolitinib was purchased from LC Laboratories, Woburn MA.

### Cell culture and qPCR

The REVEAL assay was performed as described previously [18]. In brief, resting CD4^+^ T cells (rCD4s) were isolated from peripheral blood mononuclear cells (PBMCs) obtained from aviremic HIV+ donors. Aliquots of 5 × 10^6^ rCD4s were cultured under multiple conditions: a negative control consisting of culture medium and dimethyl sulfoxide (DMSO; compound solvent), ingenol-3,20-dibenzoate (100 nM), ingenol B (100 nM), or CD3/CD28 antibody-coated magnetic beads (positive control). At 72 h, real time quantitative polymerase chain reaction (qPCR) was performed on culture supernatant to quantify viral release from rCD4 cells. In order to evaluate cytokine release from PBMCs, five million PBMCs were cultured in 1 mL RPMI-based culture media supplemented with 10% fetal calf serum. At 72 h culture supernatant was harvested and spun down to remove cellular debris. 350 μL of cell-free supernatant was processed for cytokine quantification described below.

### Cytokine measurement

Cytokines were measured in 350 μL aliquots of in vitro culture supernatant by means of a commercially available quantitative multiplex bead assay performed by the clinical reference laboratory associated with the University of Utah Medical Center, ARUP Laboratories. ARUP Cytokine Panel 12 was developed and performance characteristics determined by ARUP laboratories for evaluation of immune, infectious or inflammatory disorders in clinical or research settings, and measures the following cytokines: soluble interleukin 2 receptor (CD25), interleukin 12 (IL-12), interferon gamma (IFNγ), interleukin 4 (IL-4), interleukin 5 (IL-5), interleukin 10 (IL-10), interleukin 13 (IL-13), interleukin 1 beta (IL-1β), interleukin 6 (IL-6), interleukin 8 (IL-8), tumor necrosis factor alpha (TNFα), and interleukin 2 (IL-2).

### PKC agonist in vitro dose–response

A dose–response experiment was conducted in order to compare the pro-inflammatory cytokine induction profiles of PKC agonists that have been shown to reactivate latent HIV in vitro (bryostatin-1, prostratin and ingenol-3,20-dibenzoate). PBMCs from aviremic HIV-1 positive donors were exposed to three concentrations of each of these PKC agonists and cytokine concentrations in culture supernatant were evaluated at 72 h using the quantitative multiplex bead assay described above. LRAs were tested at concentrations shown to be active in reversing latency as well as one log higher and one log lower than this active concentration (ingenol-3,20-dibenzoate: 10 nM/100 nM/1000 nM; bryostatin-1: 1 nM/10 nM/100 nM; prostratin: 30 nM/300 nM/3000 nM).

### Individual cell cytokine production (In vitro)

Changes in intracellular TNFα and IFNγ levels in CD4, CD8, CD11b, and CD56 positive cells were examined to determine the effect of ingenol-3,20-dibenzoate and CD3/CD28 antibody-coated magnetic beads on the induction of pro-inflammatory cytokines in individual cell types. PBMCs were isolated from the whole blood of two healthy donors using a Lymphoprep density gradient (Cat# 07861, StemCell Technologies). 5 × 10^6^ total PBMCs were cultured for 72 h in the presence or absence of ingenol-3,20-dibenzoate (100 nM) or CD3/CD28 antibody-coated magnetic beads. At 64 h post-stimulant exposure, 0.067 μL/1 × 10^5^ BD GolgiStop™ Protein Transport Inhibitor (Cat# 554724) was added to each sample to prevent cytokine release. Adherent cells were removed from the cell culture plate with StemPro^®^ Accutase^®^ cell dissociation reagent (Cat# A1110501, Gibco™) prior to treatment of total PBMCs with human FcR blocking reagent (Cat# 130-059-901, MACS Miltenyi Biotec). Cells were washed with 1× phosphate buffered saline (PBS) + 0.1% sodium azide prior to staining with 0.1 µL/1 × 10^5^ Fixable Viability Dye eFluor^®^ 450 (Cat# 65-0863-14, Affymetrix eBioscience) for 30 min at 4°. Cells were then washed with 1× PBS + 0.1% sodium azide prior to staining cells in 100 µL 1× PBS + 0.1% sodium azide plus 0.5 µL CD4 [CD4 antibody, PE (Cat# MHCD0404, Invitrogen)], 0.5 µL CD8 [PE mouse anti-human CD8 (Cat# 557086, BD Pharmingen™)], 2 µL CD11b [PE mouse anti-human CD11b/Mac-1 (Cat# 561001, BD Pharmingen™)], or 3 µL CD56 [PE anti-human CD56 (NCAM) (Cat# 31805, Biolegend^®^)] for 30 min at 4 °C. Cells were then washed and fixed with 100 µL BD Cytofix/Cytoperm ™ Fixation and Permeabilization Solution (Cat# 554722) for 30 min at 4 °C. After fixation, cells were washed with a perm/wash solution (1× PBS, 3% FBS, 0.1% Saponin, 0.05% Sodium Azide) prior to staining cells in 100 µL perm/wash solution plus 2 µL TNFα [FITC anti-human TNF-α antibody (Cat# 502906, Biolegend^®^)] or 3 µL IFNγ [Alexa Fluor^®^ 488 mouse anti-human IFN-γ antibody (Cat# 557718, BD Pharmingen™)] overnight at 4 °C. Cells were washed and re-suspended in 1× PBS prior to conducting flow cytometry [BD FACSCanto flow cytometer with FACSDiva acquisition software (Becton–Dickinson, Mountain View, CA)] and analysis with FlowJo (TreeStar Inc, Ashland, OR). Changes in TNFα and IFNγ percent positive cells (fold change) were calculated for each cell type (CD4, CD8, CD11b, CD56) in ingenol-3,20-dibenzoate- and CD3/CD28 antibody-treated cells compared to media alone.

### In vitro kinase inhibitor screening

Healthy donor PBMCs were isolated as described above. Isolated PBMCs were cultured overnight to allow monocyte adherence. Non-adherent PBMCs were then cultured in aliquots of 10^5^ cells for 1.5 h at 37 °C in the presence of compounds identified as potential reducers of cytokine release from a literature search and from a kinase inhibitor library (EMD Millipore; obtained from University of Utah Drug Discovery Core Facility). 159 compounds (Additional file 1: Table 1) were screened at a concentration of 10 µM. Aliquots were then exposed to various stimulant conditions: a negative control of culture medium and dimethyl sulfoxide (DMSO; compound solvent), a positive control consisting of medium and CD3/CD28 antibody-coated magnetic beads, and medium containing 100 nM ingenol-3,20-dibenzoate. At 40 h post-stimulant exposure, BD GolgiStop™ Protein Transport Inhibitor was added to each sample. At 48 h post-exposure cells were fixed and stained prior to flow cytometry analysis.

### In vitro screening flow cytometry

At 48 h post-stimulant exposure cells were washed with 1× PBS prior to staining with Fixable Viability Dye eFluor^®^ 450. Cells were washed with 1× PBS prior to fixation and washing with perm/wash solution. Cells were stained in 100 µL perm/wash with 0.5 µL APC anti-human IL-6 antibody (Cat# 501112, Biolegend^®^) overnight at 4 °C. Cells were washed with perm/wash solution prior to re-suspension in 1× PBS. Flow cytometry was performed as described above.

### Selection of compounds

Cellular IL-sixfold change was determined by comparing the percentage of IL-6-positive cells in ingenol-3,20-dibenzoate-treated cells (control) to kinase inhibitor + ingenol-3,20-dibenzoate-treated cells. Compounds that reduced cytokine release (‘hits’) were selected for further study based on the following criteria: reduction of IL-6 by fourfold and high cellular viability (>70%).

### Apoptosis and activation flow cytometry

After 48 h in vitro under control and ingenol exposures, aliquots of 10^5^ rCD4 cells were fixed using BD Cytofix™ Fixation Buffer (50% by volume; BD Biosciences) for 10 min at 37 °C. After incubation, DMSO (Fisher Scientific) was added to the sample to a final concentration of 10% and frozen at −80 °C. At the time of analysis, samples were thawed on ice and re-suspended in 2 mL of PBS. 500 μL aliquots were used for each staining and staining control. For acetyl-Histone H3 analysis, samples were pelleted and re-suspended in 100 μL of BD Phosflow™ Perm Buffer III (BD Biosciences, Cat# 558050) while vortexing, and incubated on ice for 30 min. Cells were then washed with PBS and incubated in 100 μL of PBS + 3% fetal bovine serum (FBS) containing 1 μL of acetyl-Histone H3-PE (Millipore, Cat# FCABS325PE) and 0.75 μL of cleaved caspase 3-AF488 (Cell Signaling Technology, Cat# 9669) for 1 h at room temperature protected from light. After incubation, cells were washed with PBS + 3% FBS and 100 μL of 2% paraformaldehyde (PFA) was added prior to flow cytometry acquisition. For CD69 analysis, samples were pelleted and re-suspended in 100 μL of BD Cytofix/Cytoperm™ (BD Biosciences, Cat# 554722) and incubated at 4 °C for 30 min. Cells were then washed with BD Perm/Wash™ (BD Biosciences, Cat# 554723) and incubated in 100 μL of Perm/Wash™ containing 1 μL of CD69-APC (Invitrogen, Cat# MHCD6905) and 0.75 μL of cleaved caspase 3-AF488 for 1 h at room temperature protected from light. After incubation, cells were washed with Perm/Wash buffer and 100 μL of 2% PFA was added prior to flow cytometry acquisition.

### Statistical analysis

**Fig. 1:**
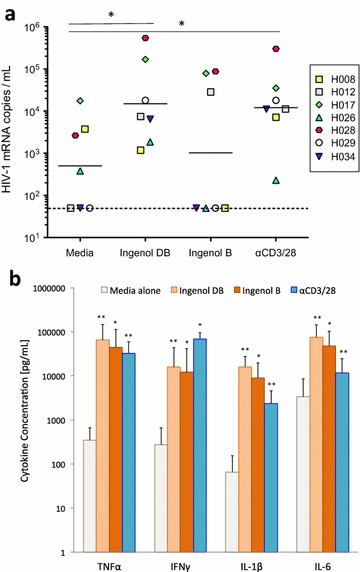
Ingenol dibenzoate reverses latency ex vivo and induces pro-inflammatory cytokine release. Ingenol-3,20-dibenzoate (ingenol DB) induced viral release from resting CD4^+^ T cells isolated from aviremic HIV-1 positive donors (n = 7; **a**) into culture supernatant to levels similar to positive control (T cell receptor stimulation via CD3 and CD28 antibodies, P value = 0.84) and significantly above the media alone condition (negative control; P value = 0.04). Ingenol B induced viral release in a minority of donor cultures and did not differ significantly from negative control (P value = 0.31). Despite differences in latency reversal, concentrations of pro-inflammatory cytokines TNFα, IFNγ, IL-1β and IL-6 were significantly elevated by both ingenol DB and ingenol B compared to negative control at 72 h in the supernatant of PBMCs from the same participants (**b**). Mean values and standard deviation are shown. Pro-inflammatory cytokine concentrations were significantly elevated in positive control cultures to levels similar to both ingenol conditions. *P value <0.05; **P value <0.01

Pair-wise comparisons of cytokine concentrations (Figs. 1b, 4) and HIV-1 viral release (Figs. 1a, 3) under different experimental and control conditions were performed using a non-parametric Wilcoxon matched-pairs signed rank test. Flow cytometric measurements of CD69 expression and activated caspase 3 (Fig. 5) were evaluated using a paired t test. Individual measurements of cytokine concentrations, flow cytometry and qPCR values were tabulated and evaluated for statistical significance using software from GraphPad Prism Version 5.0f (GraphPad Software, San Diego CA).

## Results

Ingenol-3,20-dibenzoate and ingenol B have been shown to induce proviral transcription in resting CD4^+^ T cells from aviremic participants on ART [13–15, 18], however concomitant pro-inflammatory cytokine upregulation characteristic of PKC agonists represent a significant barrier to their application as latency reversing agents in vivo. We initially sought to characterize the degree to which these compounds induce viral reactivation and pro-inflammatory cytokine release using cells obtained from aviremic participants on ART (Fig. 1). Using the REVEAL assay [18], we found that ingenol-3,20-dibenzoate (100 nM) reproducibly led to latency reversal with levels of viral release significantly above background (P value = 0.04) and similar to positive control (P value = 0.84; Fig. 1a), while ingenol B (100 nM) did not induce viral release to levels significantly above medium-alone negative control cultures (P value = 0.31).

PBMCs from the same donors were cultured with ingenol-3,20-dibenzoate or ingenol B (100 nM for both drugs) for 72 h (Fig. 1b). Pro-inflammatory cytokine concentrations were measured from cell-free culture supernatant. Compared to medium-alone controls, both ingenol-3,20-dibenzoate and ingenol B significantly increased TNFα, IFNγ, IL-1β and IL-6 to concentrations similar to those observed after T cell receptor stimulation with CD3/CD28 antibodies (positive control) in PBMCs. Pro-inflammatory cytokine production in PBMC cultures exposed to ingenol-3,20-dibenzoate was driven by CD8^+^ and CD56^+^ cells, while T cell receptor stimulation resulted in pro-inflammatory cytokine production from CD4^+^, CD8^+^, CD56^+^ and to a lesser extent CD11b+ cells (Additional file 2: Figure 1). In contrast, pro-inflammatory cytokines were not induced when purified resting CD4^+^ T cells were incubated with either ingenol species (Additional file 3: Figure 2). Ingenol-3,20-dibenzoate induced higher cytokine release than other PKC agonists tested, namely prostratin and bryostatin-1, at concentrations known to reverse latency in vitro (Additional file 4: Figure 3).

**Fig. 2:**
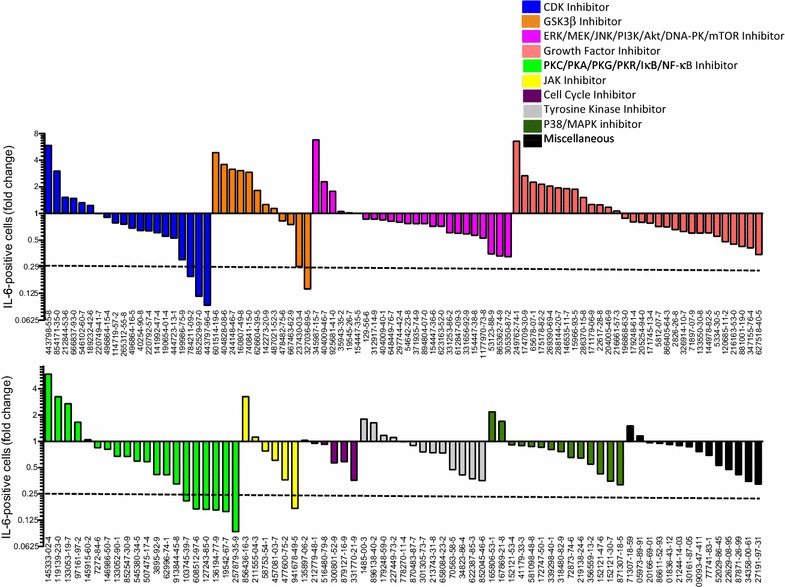
Kinase inhibitor screening identifies compounds that attenuate ingenol-induced cytokine release. Screening for changes in IL-6 production in healthy donor PBMCs exposed to ingenol-3,20-dibenzoate and 159 kinase inhibitors reveals twelve ‘hits’ that decrease intracellular IL-6 concentrations at least fourfold compared to ingenol-3,20-dibenzoate alone. Compounds are identified by Chemical Abstracts Service (CAS) number and color-coded by known function. Four of the twelve hits demonstrated high cellular viability (71–78%). Compound 941678-49-5, a Janus Kinase inhibitor, also known as ruxolitinib and FDA-approved for the treatment of myelofibrosis, was selected for additional experiments

We then conducted a small screen among known kinase inhibitors to identify compounds able to decrease pro-inflammatory cytokine release in the presence of ingenol-3,20-dibenzoate (Fig. 2). Healthy donor PBMCs were cultured with ingenol-3,20-dibenzoate (100 nM) along with compounds from a library of known kinase inhibitors. IL-6 was chosen as a representative pro-inflammatory cytokine for this screen. Intracellular IL-6 expression was measured by flow cytometry at 48 h. 159 compounds were screened in total. Twelve compounds (7.5%) reduced intracellular IL-6 expression by at least fourfold. Compound hits included three cyclin dependent kinase (CDK) inhibitors (Chemical Abstracts Service (CAS)# 784211-09-2; 852529-97-0; 443797-96-4), five protein kinase inhibitors (CAS# 103745-39-7, 608512-97-6; 127243-85-0; 136194-77-9, 257879-35-9), one inhibitor of phosphorylation of IκB (CAS# 19542-67-7), two glycogen synthase kinase 3 beta (GSK-3β) inhibitors (CAS# 237430-03-4; 327036-89-5), and a Janus kinase (JAK) inhibitor (CAS# 941678-49-5) (Fig. 2). Among the twelve compounds, eight compounds demonstrated low cellular viability (10-39% viability) and four demonstrated high cellular viability (71–78% viable; CAS# 136194-77-9, 257879-35-9, 941678-49-5, 103745-39-7). Two of these were identified as PKC inhibitors and were therefore not pursued further (CAS# 136194-77-9; 257879-35-9). The two remaining compounds included CAS# 941678-49-5, a Janus kinase inhibitor also known as ruxolitinib, and 103745-39-7, a Protein Kinase A (PKA)/Protein Kinase G (PKG) inhibitor. Ruxolitinib is FDA-approved for the treatment of myelofibrosis and was previously shown to efficiently block pro-inflammatory cytokine release from T cells in vivo in the context of myelofibrosis [31]. Ruxolitinib has favorable pharmacokinetics and a well-characterized in vivo safety profile [33], and therefore represented a promising candidate to attenuate ingenol-induced pro-inflammatory cytokine induction.

**Fig. 3:**
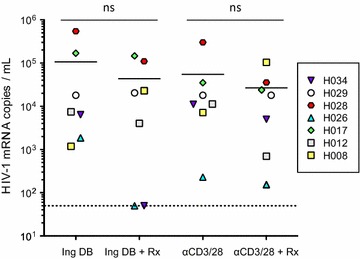
Ruxolitinib does not affect viral release from resting CD4^+^ T cells. No significant change in HIV-1 viral release was observed in resting CD4^+^ T cell cultures exposed to ingenol-3,20-dibenzoate (Ing DB) or antibodies against CD3 and CD28 (αCD3/28, positive control) in the presence of ruxolitinib (Rx) compared to Ing DB or αCD3/28 alone. *Horizontal bars* represent geometric mean values within each culture condition. Resting CD4^+^ T cells were obtained from aviremic ART-treated individuals cultured ex vivo (n = 7)

Based on these results, we performed the REVEAL assay using resting CD4^+^ T cells from aviremic participants on ART (n = 7) to evaluate whether ruxolitinib (100 nM) affected latency reversal by ingenol-3,20-dibenzoate (100 nM). Ingenol-3,20-dibenzoate was selected based on its ability to induce viral release to a similar degree to T cell receptor stimulation (Fig. 1a). Cultures exposed to ingenol-3,20-dibenzoate plus ruxolitinib demonstrated no significant change with respect to viral release compared to culture conditions not containing ingenol-3,20-dibenzoate alone (Fig. 3).

**Fig. 4:**
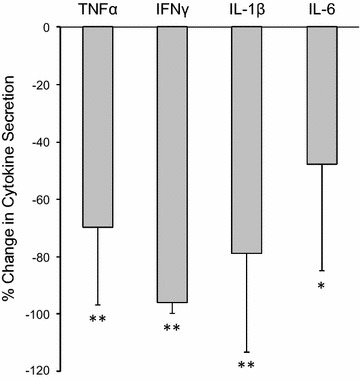
Ruxolitinib significantly decreases ingenol-induced cytokine release. *Bars* represent the median change in pro-inflammatory cytokine concentrations in PBMC cultures exposed to ruxolitinib + ingenol-3,20-dibenzoate (ingenol DB) compared to ingenol DB alone. PBMCs from aviremic ART-treated individuals (n = 7) were cultured for 72 h. One standard deviation shown by *error bars*. *P value <0.05; **P value <0.01

We then sought to test whether the ability of ruxolitinib to suppress IL-6 secretion in the context of ingenol-3,20-dibenzoate would apply to other pro-inflammatory cytokines. To that end, we tested TNFα, IFNγ, IL-1β and IL-6 secretion in cells from HIV-1-infected aviremic patients. We observed that the secretion of these pro-inflammatory cytokines (all upregulated by ingenol derivatives, Fig. 1b) decreased significantly in the presence of ruxolitinib (Fig. 4). Ruxolitinib also suppressed release of CD25 (IL-2r), IL-5 and IL-13, but did not affect levels of IL-2, IL-4, IL-8, IL-10, or IL-12 in the setting of ingenol exposure (Additional file 5: Figure 4a). Ruxolitinib demonstrated less efficacy at reducing T cell receptor stimulation-induced pro-inflammatory cytokine release (Additional file 5: Figure 4b). For example, IL-6 and IL-1β concentrations were not significantly altered by the addition of ruxolitinib to αCD3/CD28-exposed cultures (in contrast to significant reductions observed in ingenol-exposed cultures shown in Fig. 4). This may be due to the pleiotropic effects of T cell receptor stimulation on cellular activation pathways and is the subject of ongoing study.

**Fig. 5:**
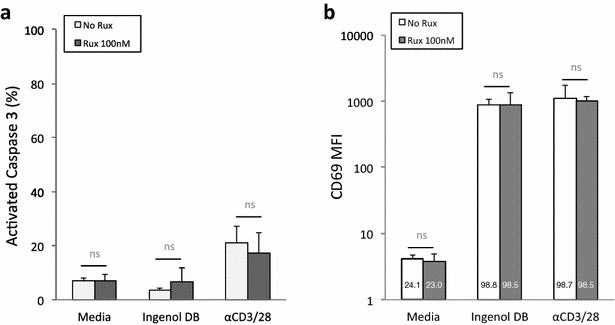
Ruxolitinib does not alter apoptosis threshold or CD69 expression in the presence of ingenol-3,20-dibenzoate. Ruxolitinib (100 nM) did not significantly alter the percentage of resting CD4^+^ T cells expressing activated caspase 3 (**a**) or expression of CD69 (represented as the mean florescence intensity or MFI with the total percentage of cells in each culture condition expressing CD69 within each *bar*; **b**) in the presence of ingenol-3,20-dibenzoate (ingenol DB). Mean values and standard deviation are shown for four independent experiments using resting CD4^+^ T cells from aviremic ART-treated participants (n = 4)

We have previously observed that CD69, a cell surface protein upregulated early in T cell activation, serves as a biomarker of PKC activation with ingenol compounds [18]. Furthermore, these compounds do not induce apoptosis in resting CD4^+^ T cells above baseline (measured by activated caspase 3) or loss of viability in general [18]. We sought to determine whether exposure to ruxolitinib would affect caspase activation or CD69 expression (Fig. 5). Addition of 100 nM ruxolitinib to resting CD4^+^ T cell cultures from aviremic participants on ART (n = 4) exposed to 100 nM ingenol-3,20-dibenzoate demonstrated no significant changes with respect to the percentage of cells expressing activated caspase 3 (Fig. 5a), CD69, or the mean florescence intensity of CD69 expression (Fig. 5b). Viability measured by forward- and side-scatter gating via flow cytometry ranged between 89 and 93% across conditions and was not statistically different between them (data not shown). We conclude that ruxolitinib does not alter the apoptosis threshold or influence CD69 expression in CD4^+^ T cells in the context of ingenol 3,20 dibenzoate exposure.

## Discussion

The earliest clinical trials attempting to perturb the HIV-1 latent reservoir in vivo made use of a strategy of global T cell activation and resulted in significant toxicity, due in part to induction of pro-inflammatory cytokines [34, 35]. Subsequent clinical trials have tested LRAs that induce minimal T cell activation including histone deacetylase inhibitors and disulfiram, however these trials did not demonstrate perturbation of the latent reservoir in vivo [11]. The modest results of these trials support the need for a different approach to latency reversal involving strong activators. In a comparative analysis across multiple in vitro latency model systems and in cells from aviremic patients ex vivo, PKC agonists were the only LRA class to consistently demonstrate latency reversal, aside from T cell receptor engagement [36].

PKC agonists, and ingenol compounds in particular, are promising candidate latency reversal agents for HIV-1 eradication [23]. However, their in vivo utility as LRAs could be compromised by induction of pro-inflammatory cytokine release common to many protein kinase C agonists. We propose a novel approach to address this potentially toxic adverse effect of an otherwise promising class of latency reversal agents. The FDA-approved drug ruxolitinib does not significantly affect the latency reversing potential of ingenol-3,20-dibenzoate ex vivo using resting CD4^+^ T cells from aviremic HIV-1 positive individuals on ART. Previously published work demonstrated that ruxolitinib inhibited latency reversal in a primary cell latency model when used at high concentrations (1 μM and greater) [37]. At 100 nM, Gavegnano et al. [37] reported only 20% inhibition of viral reactivation, whereas we observed no significant inhibition of latency reversal at that same concentration. Ruxolitinib does however effectively decrease ingenol-induced pro-inflammatory cytokine release.

In vitro cell culture assays manipulating cytokine release are imperfect models of cytokine storm in vivo. Future studies evaluating the potential role of Janus kinase inhibition in attenuating PKC-induced cytokine release in non-human in vivo HIV-1 latency models are an important next step toward furthering this potential clinical strategy. Further studies will also be required to determine the dose–response relationship between ruxolitinib and cytokine suppression in PBMCs. This in turn will inform whether ruxolitinib, at concentrations achievable in vivo, can decrease PKC-induced cytokine stimulation to levels that would allow these latency reversing agents to be tolerated in pilot clinical trials.

## Conclusion

JAK inhibition decreases ingenol-induced pro-inflammatory cytokine release without significantly reducing latency reversal in resting CD4^+^ T cells from aviremic HIV-1 positive patients on ART ex vivo. Co-administration of the FDA-approved Janus kinase inhibitor ruxolitinib may help to overcome a major barrier to the use of ingenol compounds (or other PKC agonists) as latency reversal agents for pilot HIV-1 eradication clinical trials.

## Additional files

**Additional file 1: Table 1.** Compounds screened in the kinase inhibitor library. Compounds are listed by Chemical Abstracts Service (CAS) number in the order shown in Fig. 2 along with the compound’s common names, known functions, and if they were observed to reduce IC IL-6 levels by ≥fourfold.

**Additional file 2: Figure 1.** Ingenol-3,20-dibenzoate (Ingenol DB) and CD3/CD28 antibody-induced fold changes in TNFα and IFNγ among peripheral blood cell subsets. Ingenol DB and CD3/28 antibodies increased the number of TNFα and IFNγ positive CD8^+^ and CD56^+^ cells compared to media alone at 72 h post-stimulation in two donors. CD3/CD28 antibody treatment increased the number of TNFα and IFNγ positive CD4^+^ cells, whereas ingenol DB treatment decreased the number of cytokine positive cells in both donors, likely due to a down-regulation of CD4 upon ingenol DB treatment [30]. TNFα and IFNγ positive CD11b^+^ cells varied between donors.

**Additional file 3: Figure 2.** Ingenols do not induce pro-inflammatory cytokine release in purified resting CD4^+^ T cell cultures. Concentrations of pro-inflammatory cytokines TNFα, IFNγ, IL-1β and IL-6 were not significantly elevated in the presence of ingenol-3,20-dibenzoate (ingenol DB) or ingenol B compared to media alone control at 72 h in purified resting CD4^+^ T cell cultures. Mean values and standard deviation of seven independent experiments using resting CD4^+^ T cells from aviremic ART-treated HIV positive participants are shown. Pro-inflammatory cytokine concentrations were significantly elevated in positive control cultures (T cell receptor stimulation via CD3 and CD28 antibodies). ** P value <0.01; *** P value <0.001.

**Additional file 4: Figure 3.** PKC agonists induce pro-inflammatory cytokines in a dose-dependent manner. PBMCs from aviremic, HIV-1-infected participants (n = 4) were exposed to PKC agonists ingenol-3,20-dibenzoate, prostratin and bryostatin-1 at concentrations shown to reverse latency as well as tenfold higher and lower concentrations. Pro-inflammatory cytokine concentrations were measured in culture supernatant after 72 h in culture. Bars represent mean fold-change above cytokine concentrations from media-alone PBMC cultures (negative control) with one standard deviation shown. When comparing concentrations of these PKC agonists previously shown to reverse viral latency, ingenol-3,20-dibenzoate demonstrated the highest pro-inflammatory cytokine induction.

**Additional file 5: Figure 4.** Ruxolitinib-induced cytokine changes in PBMCs from aviremic HIV-1 infected donors. CD25 (IL2r), IL-5, and IL-13 concentrations were significantly decreased in PBMC cultures exposed to ruxolitinib + ingenol-3,20-dibenzoate compared to ingenol-3,20-dibenzoate alone. Ruxolitinib significantly reduced these cytokine concentrations in CD3/28 antibody-treated PBMC cultures as well, along with reduction in IL-10. Ruxolitinib did not decrease pro-inflammatory cytokines induced by CD3/28 antibody stimulation to the same degree as ingenol-stimulated cells (shown in Fig. 4).
