# Supplementary figures and images for: Janus kinase inhibition suppresses PKC-induced cytokine release without affecting HIV-1 latency reversal ex vivo
Source: Retrovirology. 2016 Dec 20;13:88. doi: 10.1186/s12977-016-0319-0 (PMC5175306; doi:10.1186/s12977-016-0319-0)

## Slide 1
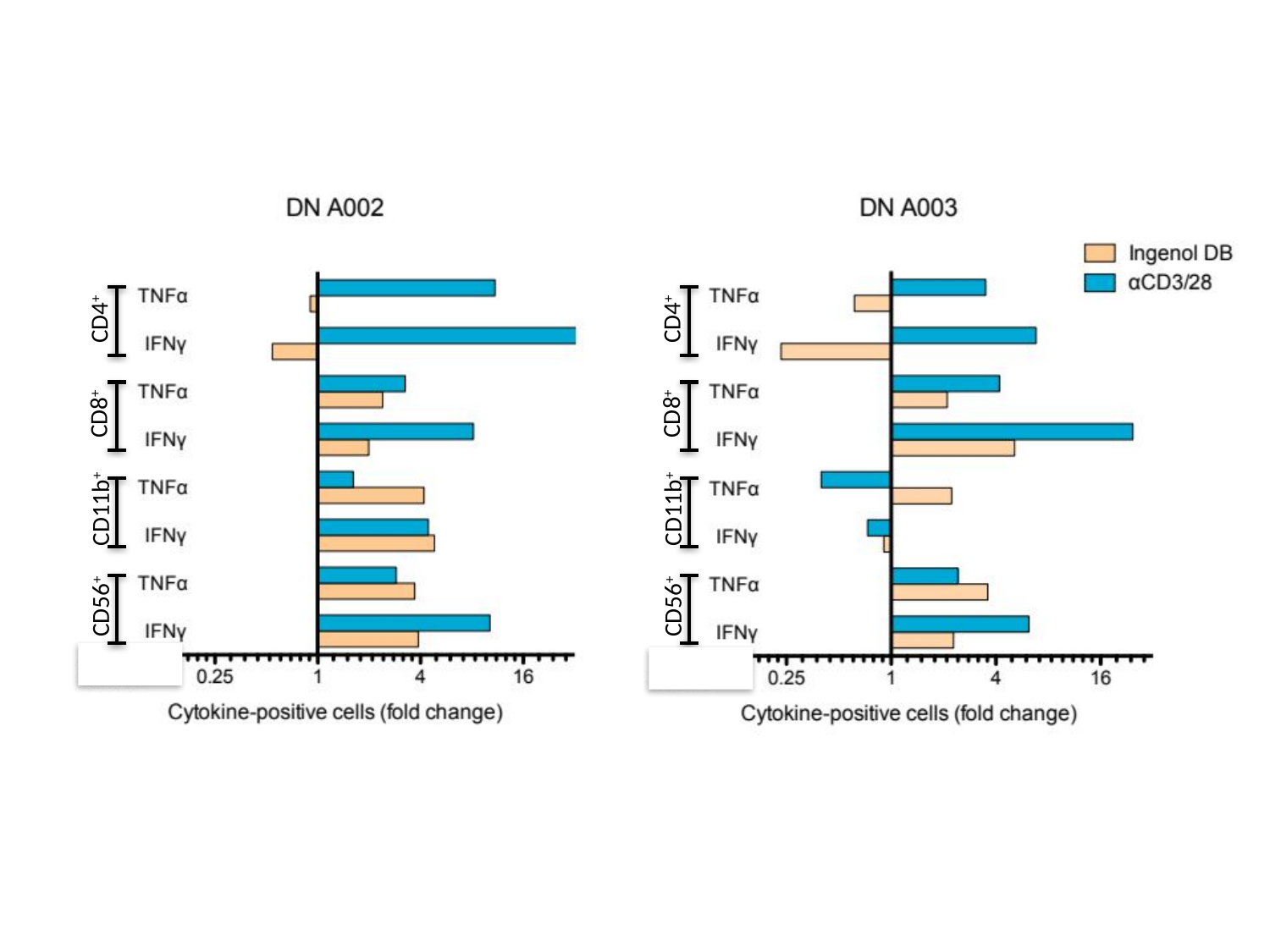

CD4+
CD8+
CD11b+
CD56+
CD4+
CD8+
CD11b+
CD56+

Supplement: Supplementary file 2 — Additional file 2: Figure 1. Ingenol-3,20-dibenzoate (Ingenol DB) and CD3/CD28 antibody-induced fold changes in TNFα and IFNγ among peripheral blood cell subsets. Ingenol DB and CD3/28 antibodies increased the number of TNFα and IFNγ positive CD8+ and CD56+ cells compared to media alone at 72 h post-stimulation in two donors. CD3/CD28 antibody treatment increased the number of TNFα and IFNγ positive CD4+ cells, whereas ingenol DB treatment decreased the number of cytokine positive cells in both donors, likely due to a down-regulation of CD4 upon ingenol DB treatment [30]. TNFα and IFNγ positive CD11b+ cells varied between donors. [file 12977_2016_319_MOESM2_ESM.pptx]

## Slide 1
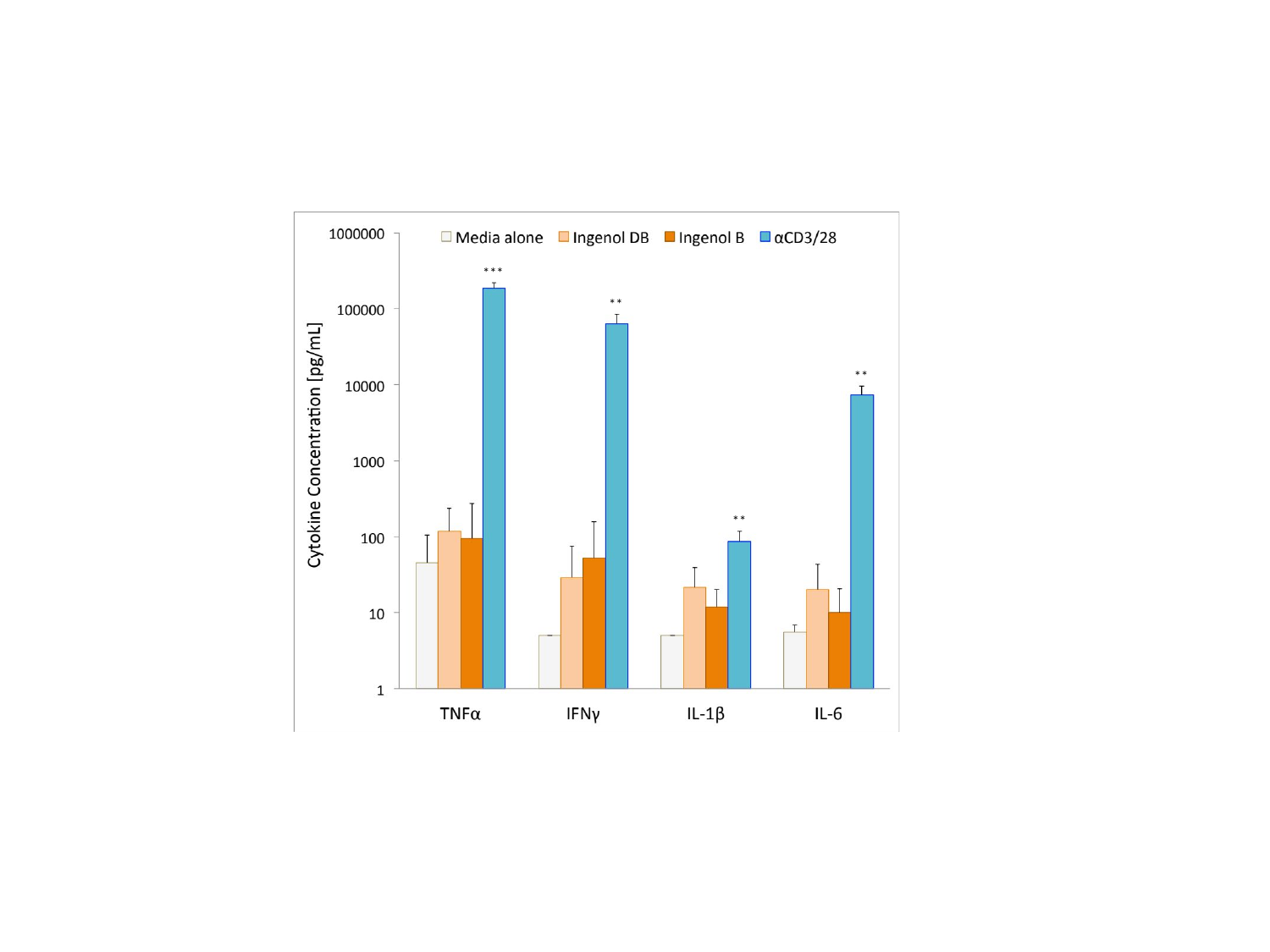

***
**
**
**

Supplement: Supplementary file 3 — Additional file 3: Figure 2. Ingenols do not induce pro-inflammatory cytokine release in purified resting CD4+ T cell cultures. Concentrations of pro-inflammatory cytokines TNFα, IFNγ, IL-1β and IL-6 were not significantly elevated in the presence of ingenol-3,20-dibenzoate (ingenol DB) or ingenol B compared to media alone control at 72 h in purified resting CD4+ T cell cultures. Mean values and standard deviation of seven independent experiments using resting CD4+ T cells from aviremic ART-treated HIV positive participants are shown. Pro-inflammatory cytokine concentrations were significantly elevated in positive control cultures (T cell receptor stimulation via CD3 and CD28 antibodies). ** P value <0.01; *** P value <0.001. [file 12977_2016_319_MOESM3_ESM.pptx]

## Slide 1
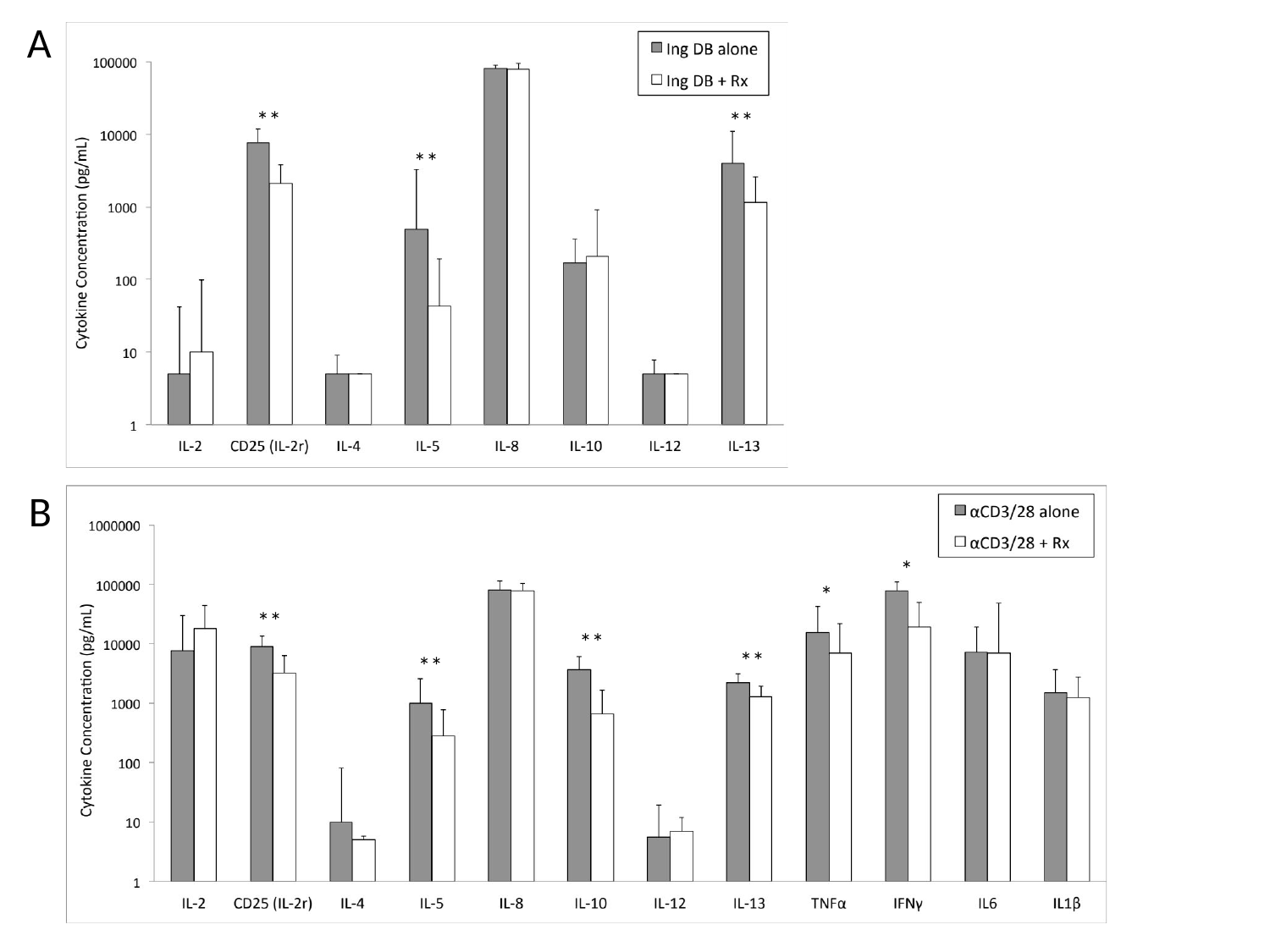

A
**
**
**
B
*
*
**
**
**
**

Supplement: Supplementary file 5 — Additional file 5: Figure 4. Ruxolitinib-induced cytokine changes in PBMCs from aviremic HIV-1 infected donors. CD25 (IL2r), IL-5, and IL-13 concentrations were significantly decreased in PBMC cultures exposed to ruxolitinib + ingenol-3,20-dibenzoate compared to ingenol-3,20-dibenzoate alone. Ruxolitinib significantly reduced these cytokine concentrations in CD3/28 antibody-treated PBMC cultures as well, along with reduction in IL-10. Ruxolitinib did not decrease pro-inflammatory cytokines induced by CD3/28 antibody stimulation to the same degree as ingenol-stimulated cells (shown in Fig. 4). [file 12977_2016_319_MOESM5_ESM.pptx]
