# Supplementary material for: Janus kinase inhibition suppresses PKC-induced cytokine release without affecting HIV-1 latency reversal ex vivo
Source: Retrovirology. 2016 Dec 20;13:88. doi: 10.1186/s12977-016-0319-0 (PMC5175306; doi:10.1186/s12977-016-0319-0)
Supplement: Supplementary file 4 — Additional file 4: Figure 3. PKC agonists induce pro-inflammatory cytokines in a dose-dependent manner. PBMCs from aviremic, HIV-1-infected participants (n = 4) were exposed to PKC agonists ingenol-3,20-dibenzoate, prostratin and bryostatin-1 at concentrations shown to reverse latency as well as tenfold higher and lower concentrations. Pro-inflammatory cytokine concentrations were measured in culture supernatant after 72 h in culture. Bars represent mean fold-change above cytokine concentrations from media-alone PBMC cultures (negative control) with one standard deviation shown. When comparing concentrations of these PKC agonists previously shown to reverse viral latency, ingenol-3,20-dibenzoate demonstrated the highest pro-inflammatory cytokine induction. [file 12977_2016_319_MOESM4_ESM.pptx]

## Slide 1
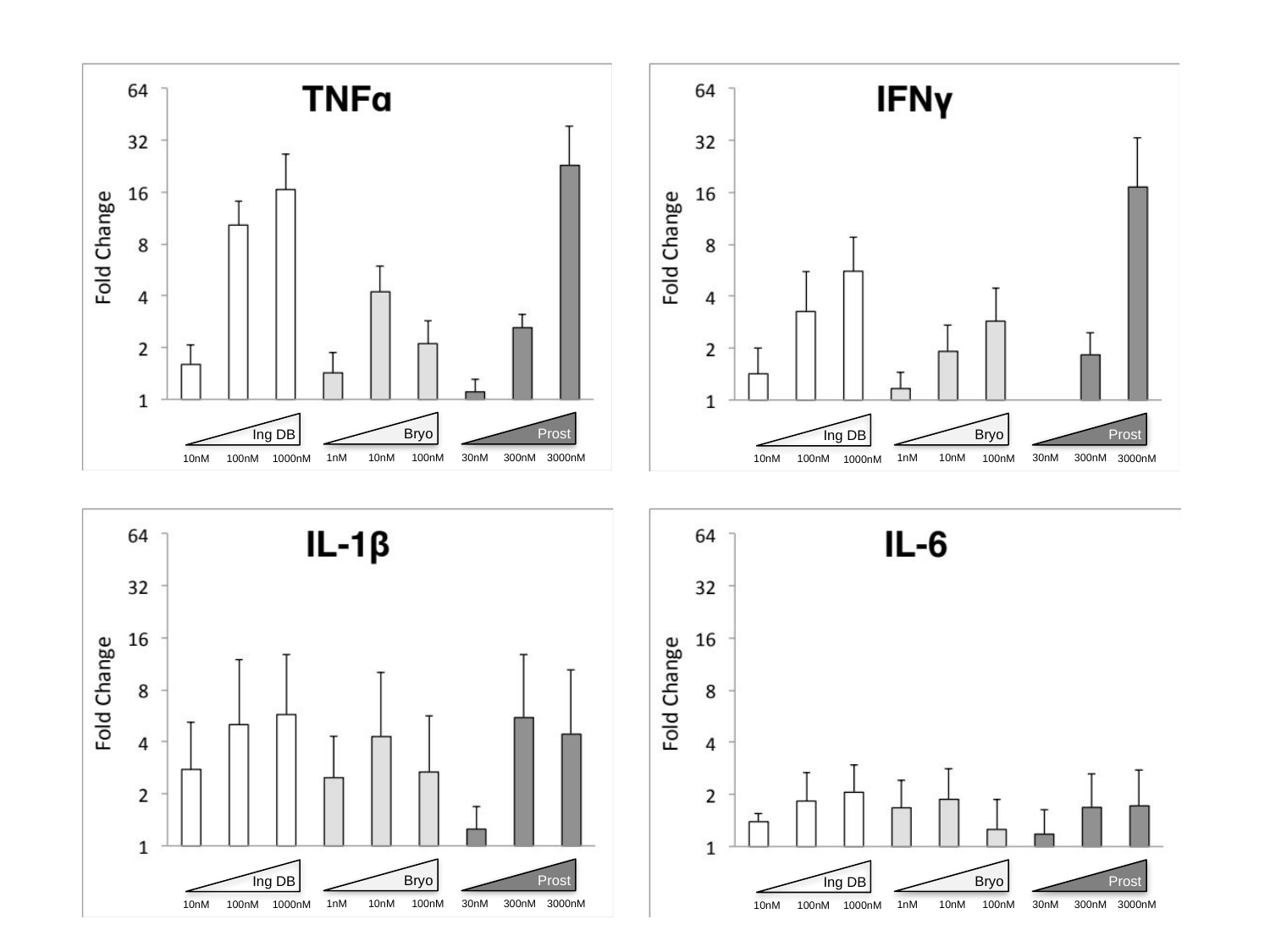

Bryo
Prost
Bryo
Prost
Ing DB
Ing DB
1nM
10nM
30nM
300nM
100nM
3000nM
1nM
10nM
30nM
300nM
10nM
100nM
100nM
3000nM
1000nM
10nM
100nM
1000nM
Bryo
Prost
Bryo
Prost
Ing DB
Ing DB
1nM
10nM
30nM
300nM
100nM
3000nM
1nM
10nM
30nM
300nM
10nM
100nM
100nM
3000nM
1000nM
10nM
100nM
1000nM
